# Caring for Infants with Robin Sequence Treated with the Tübingen Palatal Plate: A Review of Personal Practice

**DOI:** 10.3390/children10101628

**Published:** 2023-09-29

**Authors:** Petra Knechtel, Christina Weismann, Christian F. Poets

**Affiliations:** 1 Department of Neonatology, Tübingen University Hospital, 72076 Tübingen, Germany; 2 Center for Cleft Palate and Craniofacial Malformations, Tübingen University Hospital, 72076 Tübingen, Germany; 3 Department of Orthodontics, Tübingen University Hospital, 72076 Tübingen, Germany

**Keywords:** Pierre Robin sequence, intraoral scanning, functional treatment, feeding, cleft palate, upper airway obstruction, mandibular retrognathia

## Abstract

The Tübingen Palatal Plate (TPP) is a minimally invasive yet highly effective functional orthodontic treatment for upper airway obstruction in infants with Robin Sequence (RS). It consists of a palatal plate to cover the cleft and a velar extension that shifts the root of the tongue forward. We review our practical experience with this approach. First, upon admission, our local orthodontists perform an (3-D) intraoral scan of the maxilla. Based on the scan data, the TPP is manufactured in a semi-digital workflow. The length and angulation of its extension is checked via awake laryngoscopy and the effectiveness confirmed by a sleep study. Plates are kept in place by adhesive cream. When inserting the TPP, the tip of the tongue must be visible. Next, metal fixation bows should be secured to the forehead using tape and elastic bands. Plates are removed daily for cleaning, and the oral mucosa is then checked for pressure marks. Feeding training (initially only via finger feeding) may even start before plate insertion. Breathing often normalizes immediately once the plate is inserted. For isolated RS, we have never had to perform a tracheostomy. This has largely been possible through our highly dedicated and competent team, particularly the nursing staff, and the early involvement of parents.

## 1. Introduction

Robin sequence (RS), previously called Pierre Robin sequence, is a congenital disorder first described by the French stomatologist Pierre Robin in 1923 as consisting of retrognathia and glossoptosis, both resulting in upper airway obstruction, failure to thrive, mental retardation, and even sudden death [1]. We prefer the term RS, as the use of first names is unusual in eponyms [2,3]. In about half the cases, RS is associated with other, i.e., syndromic, conditions [4,5]. It affects about 1 in 5250–14,000 neonates [6,7]. The main treatment goals are to prevent the tongue from falling back, thereby avoiding potentially life-threatening hypoxemia, and to enable affected infants to be fed fully orally, which otherwise constitutes a major hurdle. These goals may be achieved via surgical (e.g., mandibular distraction osteogenesis (MDO) or tongue–lip adhesion [8,9]) or conservative (e.g., using palatal plates or a nasopharyngeal airway [10,11]) approaches. As 80–90% of RS infants also have a cleft palate, palatal plates, similar to those used for nasoalveolar molding [12], are often used to facilitate sucking. About 25 years ago, we modified such plates so that they also facilitated breathing. For the first 20 years of our program, we obtained a maxillary imprint and manually designed the plate based on casts made of compound soft and hard acrylic (Forestacryl-Strong-S, Foerster; Germany), which carried a risk of aspiration and was unpleasant to the infant. Since 2018, we have moved to a digital workflow, including intraoral scanning of the maxilla, which infants tolerate much better (see below). Here, we discuss the practical aspects of applying this functional yet highly effective treatment for infants with RS.

## 2. Materials and Methods

**Figure 1:**
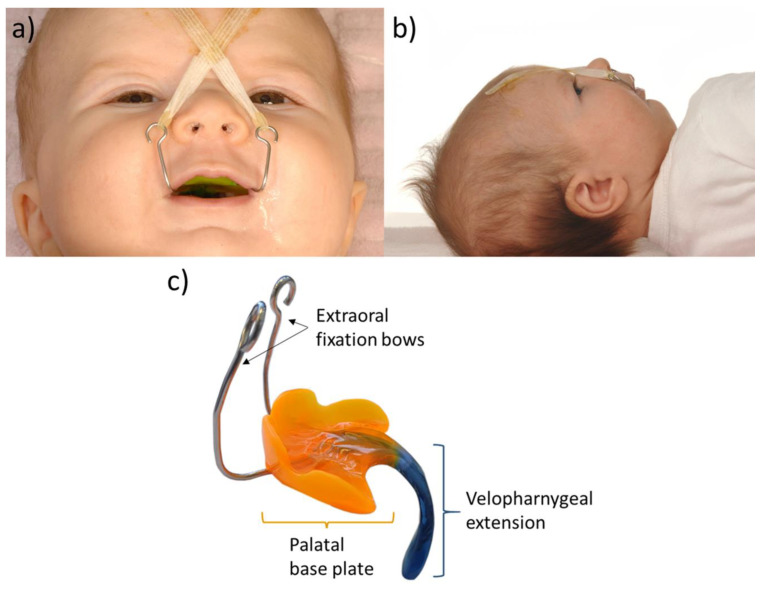
(**a**) RS patient with inserted TPP and extraoral adhesive tapes; (**b**) profile picture of a patient with RS and TPP showing the mandibular retrognathia; (**c**) example of a TPP with its characteristic velopharyngeal extension (blue) attached to a standard palatal base plate (orange) that covers the cleft. The two metal fixation bows are attached to the forehead using tape to improve plate retention, and a wire inside the extension ensures no breakage occurs that might cause aspiration. The extension is colored blue to make it easily visible against the mucosa during endoscopy.

The Tübingen Palatal Plate (TPP) was invented in our center by the orthodontist Dr. Margit Bacher in 1997. It consists of a standard palatal plate, as used in infants with a cleft palate, with an extension attached to its palatal base plate that is individually adapted to the infant’s anatomy. The base has a holding rim and incorporates two extraoral fixation bows for securing on the infant’s face, to counterbalance the pressure exerted by the tongue on the velopharyngeal extension and to prevent the plate from being leveraged off the palate (Figure 1). It stabilizes the airway without surgical intervention and supports the mandible in coming forward.

Our hospital offers care for both, children who have already been diagnosed with RS in utero and those who are transferred postnatally from other hospitals. The sooner the diagnosis of RS is established, the faster and easier the plate can be fitted and inserted, the less the child will experience situations of respiratory distress and hypoxemia, and the sooner he/she will learn to feed by him/herself. While the rationale for this treatment and its effectiveness have been extensively reported [10,13,14], this is less true for the practical aspects of applying this treatment in the neonatal unit.

## 3. Results and Practice

### 3.1. Admission

Upon admission, breathing is initially stabilized by placing the infant in the prone position. In case of extreme mandibular retrognathia, the head is even positioned at a lower level than the thorax, or a nasopharyngeal tube is inserted. If that does not suffice, the infant will receive high-flow nasal cannula (HFNC) or continuous positive airway pressure (CPAP).

After the infant has adapted to the new environment, usually during the second night after admission, a sleep study (poly(somno)graphy) is performed to determine the extent of the obstructive sleep apnea. In the course of treatment, sleep studies will be repeated to check the TPP’s effectiveness.

### 3.2. Manufacturing of the TPP

Since 2018, we have manufactured the TPP based on a digital intraoral (3D) scan (IOS) using Trios 3 or 4 (3Shape A/S, Copenhagen, Denmark). This procedure is risk-free, does not require sedation, intubation or fasting, and can be interrupted at any time (e.g., during crying). With these data, a semi-digital workflow using computer-aided design (CAD) and computer-aided manufacturing (CAM) technologies is started to produce the TPP. After a suitable IOS is obtained, the TPP is virtually designed and the data fed into a 3D printer (Solflex 170, Way2Production, Vienna, Austria) using a class IIa splint material (Freeprint Tray, VOCO, Cuxhaven, Germany), to manufacture an individual TPP in our hospital’s department of orthodontics. Others have reported a similar approach, albeit only for infants with palatal clefts and not with RS [15].

Once the plate is delivered, it is inserted without adhesive cream and an endoscopy is performed to ensure the extension is positioned exactly as required in terms of length, angle of inclination, diameter, and width to fulfill its task of preventing the tongue from falling back and supporting tongue advancement. If a suitable position is found for the extension, the definitive TPP is manufactured using conventional methods in the dental laboratory of the department of orthodontics. First, a duplicate of the TPP is made and then a cold polymerizing polymethylmethacrylate (Orthocryl Clear, Dentaurum GmbH & Co. KG, Ispringen, Germany) is used. Afterwards, the two extraoral fixation bows and the safety wire are added.

**Figure 2:**
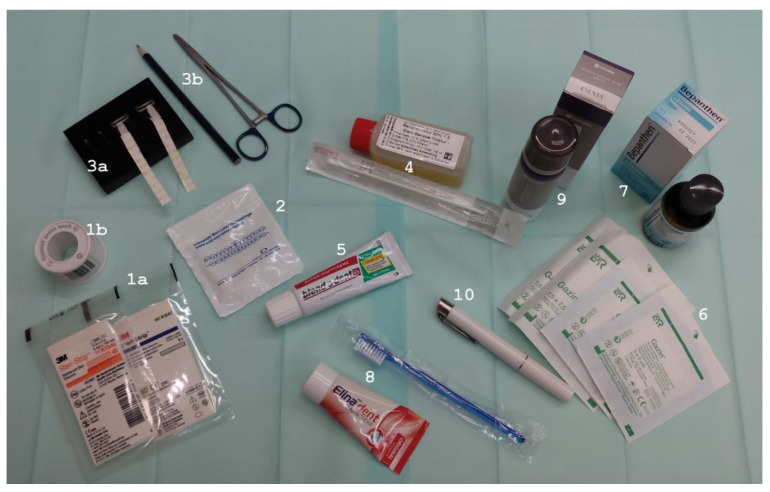
Materials needed for inserting and removing the TPP: Two adhesive tapes, e.g., (1a) Steri-Strip (3M Health Care, Saint Paul, MN, USA) or (1b) special tape for infants with hypersensitive skin, width depending on the required traction; (2) two orthodontic elastic bands, stretched over two nails hammered into a wooden board (3a), with the end of one tape being poked through an elastic band, bent back, and then fixed (3b); (4) adhesive enhancer, e.g., benzoin tincture 90% 1:5; (5) adhesive cream; (6) gauze swabs for oral care; (7) dexpanthenol solution; (8) adhesive releaser; (9) toothbrush and toothpaste; (10) small flashlight.

For inserting the plate, the following materials are prepared (Figure 2):

- Two adhesive tapes (e.g., Steri-Strip, 3M Health Care, MN, USA) or special tapes for infants with hypersensitive skin, with their width depending on the required traction.
- Two orthodontic elastic bands, stretched over two nails hammered into a wooden board, with the end of one tape being poked through an elastic band, then bent back and fixed.
- Adhesive enhancer, e.g., benzoin tincture 90% 1:5 (Caelo, Hilden, Germany).
- Adhesive cream (Procter & Gamble, Schwalbach, Germany).
- Gauze swabs for oral care.
- Dexpanthenol solution (at home, sage tea may be used instead).
- Adhesive releaser, e.g., Convatec Inc., Deeside, UK.
- Toothbrush and toothpaste.
- Small flashlight.

### 3.3. Inserting the TPP

Once endoscopy confirms the plate’s fit, and after careful preparation of the above mentioned aids, the TPP is inserted. To avoid vomiting, any endoscopy or plate insertion is performed at least 2 h after a meal. For plate insertion, parents may want to swaddle their baby in a cloth or towel to prevent his/her hands from getting in the way.

Benzoin tincture is applied to the infant’s forehead as an adhesive enhancer—the skin on the forehead needs to be dry (any sweat must be wiped off), otherwise the tapes will not stick despite the adhesive enhancer. The applied tincture must dry completely, which takes several minutes. Applying this tincture only once is sufficient as long as it is done thoroughly; we suggest to apply the fluid in the direction away from the eyes using a standard swab.

Only then is adhesive cream applied to the TPP, extending approximately one inch on, and laterally of, the frontal part of the plate, but not in its center, and not in one continuous strand to prevent cream from oozing through the gap in the middle of the holding rim. Infants with RS are often very sensitive or even traumatized in their perioral region; thus, any manipulation in this area, such as the need to remove excessive adhesive cream, should be kept to a minimum.

The amount of adhesive cream is determined by the effectiveness of keeping the plate in place: in case it almost cannot be removed the next day, too much cream has been used; if the plate comes off already after a few hours, too little cream has been used. The plate should stick for approximately 24 h. In the hospital setting, establishing a daily routine of handling the plate has proven beneficial (and even more so after discharge home). It should be kept in mind that some brands come with rather large openings of their tubes, making it somewhat difficult to use the adhesive cream as sparingly as is necessary.

Once adhesive cream has been applied, the plate is inserted by placing the tip of the extension above the tongue or as far as possible in the oral cavity, quickly followed by sliding the base of the plate forward as if turning around a corner, all the while making sure the extension remains above the tongue. The TPP should then be held firmly against the maxilla for between 20 s and several minutes, depending on the cooperation of the baby and the pressure exerted by his/her tongue. 

The specific method of inserting the plate depends on what the plate looks like as, of course, each plate is as individual as the patient—no two plates are alike; also, each nurse will develop his/her own, slightly different, method.

Once the plate is inserted, it is crucial to check that the tongue or at least its tip can still be seen. The rim of the plate is indented at the expected position of the frenulum, to avoid exerting any pressure on this sensitive structure and to allow for its movements.

The older the infant upon initiation of TPP treatment, the more resistance can be expected and the longer it takes the infant to get used to the plate, as he/she is likely to try to, and often can, lever off the plate with the base of his/her tongue. In contrast, if the baby responds only very little to the inserted plate, this may indicate that it either fits well or that its extension has not yet shifted the base of the tongue sufficiently forward.

Next, the prepared tapes are hooked into the eyelets of the TPP’s bows. Ideally, there is a second person to help with hooking and affixing the strips; these are pulled crosswise with taut tension from the extraoral bows towards the forehead and affixed where the benzoin tincture that was applied earlier will now have dried (Figure 1a).

Visible folds on the forehead are to be expected due to the tension, which can be up to a level where the infant almost cannot look out of their eyes. This is not a problem for the baby, and the parents quickly become accustomed to it, especially once they become aware of the effect the plate has on their baby’s breathing.

### 3.4. Positioning the Baby

In the first two days after fitting the initial plate, the infant should still be placed in the prone position as gravity may provoke the tongue to generate pressure marks on its own. After that time, lateral positioning is recommended for a few more days before switching the infant to the supine position as recommended for the prevention of Sudden Infant Death syndrome (SIDS).

### 3.5. Daily Oral Care and Plate Hygiene

**Figure 3:**
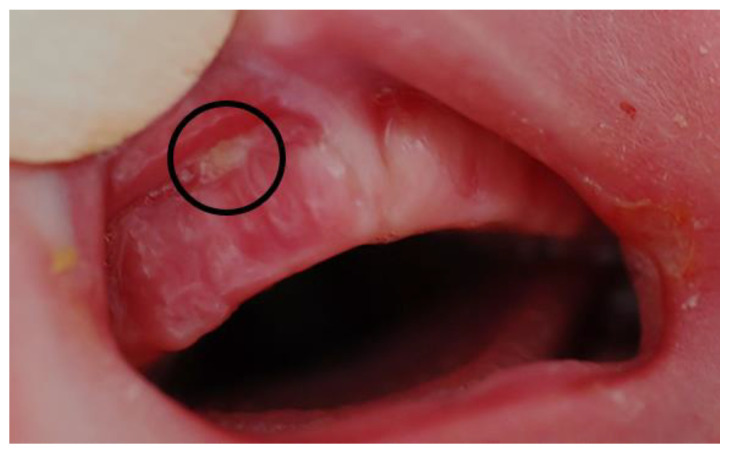
Pressure mark at the mucco-buccal fold of a patient with RS with its typical appearance: a round/oval shaped white spot with a red edge.

The TPP is removed and reinserted daily, or even twice a day if the mucosa is very sensitive, to identify any pressure marks early enough (Figure 3).

To remove the TPP, the tapes need to be unhinged; here, it is important to secure them with a fingertip where the two strips meet to avoid pulling them off accidentally and thereby causing skin lesions. The plate is snatched with thumb and index finger next to the gap, pulling it off the maxilla with gentle force and then removing it quickly. It is cleaned with a regular toothbrush and toothpaste to remove mucus and any residue of the adhesive cream while rinsing it under tap water. After that, the plate is dried, impregnated with mucosal disinfectant, and rinsed again. The alveolar ridge and palate are cleaned with gauze swabs saturated with dexpanthenol solution (after discharge, parents, may use sage tea instead). If there are neither dents nor pressure marks (a small flashlight is recommended to detect these), adhesive cream is applied to the plate, and the plate is inserted again.

The ends of the adhesive strips are hooked into the eyelets at the end of the bows, and again, it is important to secure these strips where they meet. When the traction provided by the strips is no longer sufficient, they need to be replaced. If pressure marks or lesions have occurred, the plate is not inserted again and the orthodontist on call is informed so that the plate can be abraded accordingly. This occurs usually only during the first days of TPP treatment.

## 4. Problems Potentially Necessitating Modifications of the TPP

There are several potential caveats with TPP treatment; these will be discussed in the following sections.

### 4.1. Persisting Upper Airway Obstruction

The plate needs to be modified if the infant still exhibits snoring or noisy breathing, even if only intermittently, as this suggests a problem in the region of the clefted uvula. It also requires modification if breathing is still laborious, as this may be caused by a contracting or collapsing pharyngeal wall, suggesting that the angle of the extension is not yet optimal or the extension is too narrow, too short, or too long. If required by the infant’s anatomy, the plate may also be modified by adding a ring or a short canal to the extension, or even a special mouthpiece (“flute”) [16].

### 4.2. Swallowing Problems

Not every endoscopically well-fitting plate is tolerated by the baby and may still cause problems:

- If the mouth opening is not yet sufficient, milk cannot be swallowed properly; this is mostly the case in infants with a cleft palate who were used to positioning their tongue inside the cleft during the fetal period, which is not possible anymore once the plate is inserted. In these infants, the tongue will push against the plate, the child’s swallow is empty, and milk remains in the oral cavity until infants become accustomed to the plate.
- If the angle of the extension is too steep, the child will also not swallow. When feeding is attempted, the tongue does not have enough space to perform the swallowing act and is just being pushed towards the plate. Therefore, swallowing would consequently leverage the plate off.
- If the airway becomes obstructed during swallowing, the angle of the extension is generally too flat, or the extension is not steep enough in its lower third.
- If the extension is too narrow, the tongue is pushing around the extension and/or the velum extends behind the extension.

### 4.3. Pressure Marks, Dents, and Malpositioning of the Plate

Pressure marks (Figure 3) occur if the rim of the TPP is too long or does not yet fit the maxilla, particularly in regions with a flexible oral mucosa, e.g., at the end of the tuber region, in the muco-buccal fold, or at the frenulum. Another typical location for pressure marks is on the vomer or at the palate, indicating that the base of the plate or the curvature of the extension is too high. In case the extension is too wide, pressure marks may occur at the edge of the cleft mucosa. Pressure marks may also occur if feeding training is too intense, this is due to the increased movements of the TPP during feeding.

Dents or malpositioning of the plate occur when the angle of the extension is too steep. As a result, the base of the tongue is pushing too strongly against the extension and the plate is veering off. Due to the physiological growth of the patient, dents almost always occur, particularly in the anterior region of the maxilla. These are often not painful, but we usually respond to them by adjusting the plate.

### 4.4. Problems with Too Much or Too Little Saliva

The age at treatment onset plays a role in the adaptability of the body to the “foreign object”, i.e., older infants will produce as much saliva as possible to rinse off the foreign object until they become accustomed to it; any excessive salivation can be reduced by small doses of scopolamine.

### 4.5. Nursing Problems with the TPP

- Improper insertion of the plate is an issue. If the fixation or width of the aforementioned strips are not appropriate, they provide too little traction and the plate slips off, or dents on the frontal alveolar ridge develop; if too much traction is used, pressure marks may occur on the alveolar ridge or palate.
- If too little adhesive cream has been applied, the plate wobbles on the maxilla; if too much has been used, cream may ooze into the cleft and hinder nasal breathing, or the plate cannot stick in the first place if too much cream has been applied. If the oversupply oozes through the gap in the holding rim, it must be removed, which causes unnecessary stress for the baby.
- Skin problems can be caused by the improper removal of the strips, creating redness, possibly blisters, or even skin lesions.

It is very important not to simply pull off the strips, especially since adhesive enhancer has been used. They need to be saturated well with adhesive releaser, which allows them to be taken off very easily and gently. Changing the tapes is only necessary when traction becomes insufficient.

### 4.6. Feeding

#### 4.6.1. Feeding before Plate Fitting

Feeding training starts soon after admission, i.e., even before a plate has been fitted, and depends on the respiratory condition of the baby. However, as already mentioned, even a baby with severe breathing problems has a natural desire to suck and swallow. The baby should be positioned comfortably and in a well-supported way in a semi-upright position on the nurse’s lap, as the nurse needs both hands during feeding.

The nurse offers his/her little finger with the palm downwards, so that he/she is reaching the tip of the tongue, thereby stimulating it to come forward. Depending on how weak or strong the sucking or swallowing movements are, small amounts (at first only drops) can be offered through a soft feeding device attached to a syringe—this may, in the beginning, just be a 1 mL syringe to ensure there is not too much liquid offered at once. This is performed very slowly throughout, concentrating fully on what the baby is doing, and whether it is responding to the stimulation offered and, importantly, making sure that the tip of the tongue is being reached continuously. Depending on the anatomical situation, i.e., the position of the tongue and/or the extent of the mandibular retrognathia, only glucose may be offered. Nevertheless, to avoid risking aspiration, feeding is not performed if the tongue is too far back in the pharynx.

#### 4.6.2. Advanced Feeding Training—TPP and Finger Feeding

**Figure 4:**
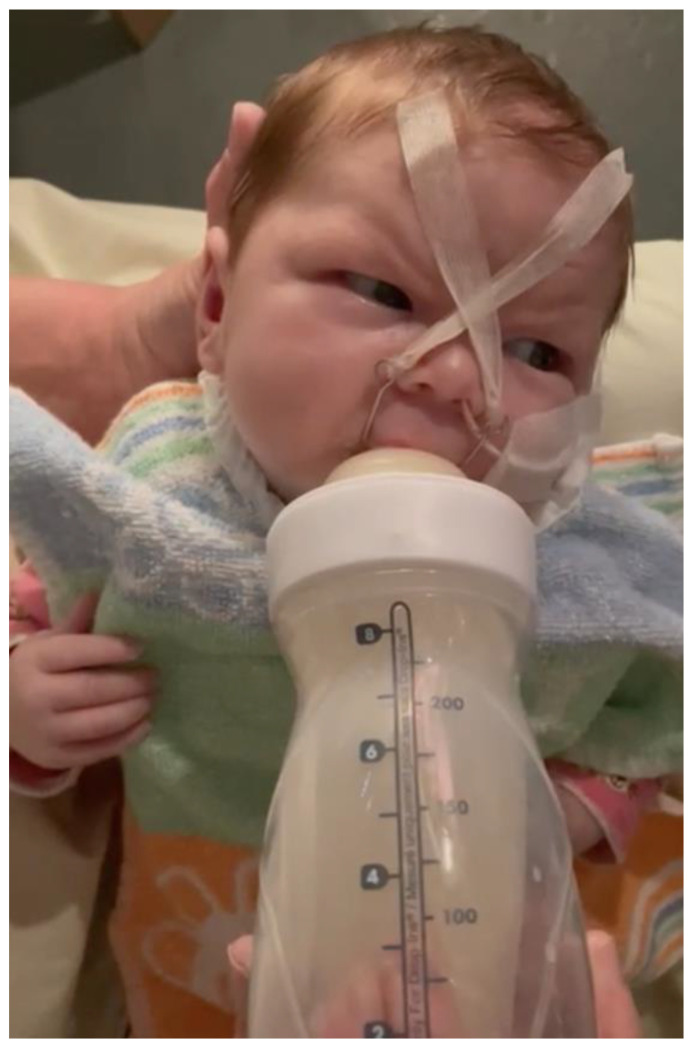
Feeding an infant with RS using the Playtex™ system. Here, the nurse can control the milk flow (photo used with parental permission).

Feeding with finger, feeder, and the TPP only makes sense once the opening of the mouth is sufficient. In general, feeding with the plate should not be attempted when pressure marks are present. With the nurse’s finger in the patient’s mouth, the forging ahead of the tongue can easily be evaluated or even triggered, and at the same time, using the syringe, milk can be administered carefully. It is beneficial to still control the position of the tongue and the swallowing capacity. This method is only used if the tongue is still too far back to begin bottle feeding (e.g., Playtex™ Baby, Shelton, CT, USA; Figure 4).

#### 4.6.3. Feeding Training with the Playtex™ System

This is a bottle system comprising a threaded tube, a soft bag (“drop-in liner”), a teat ring, and a teat. The system has proven successful for TPP-treated infants because they do not have to suck against a vacuum as is necessary with a rigid bottle.

An indispensable prerequisite for bottle feeding is the safeguarding and ongoing control that the tongue is and remains well underneath the teat when sucking, otherwise the teat will counterproductively push the tongue backwards; also, coordinated swallowing should be possible. The Playtex™ system offers the option of controlling the flow of milk so that it can be adjusted to the infant’s individual strength of sucking and swallowing. There are three hole sizes to choose from for the benefit of the individual infant.

#### 4.6.4. Course of Feeding Progress

It often takes 3–4 weeks before any transition can be made at all from finger feeding to training with the Playtex™ bottle—only rarely can a successful start with the Playtex™ system already be made within the first ten days of treatment.

Since sucking from the Playtex™ bottle requires more effort (a desired training effect for the tongue) than the much easier sucking during finger feeding, the infant will initially not be able to drink the same amount of milk as with finger feeding.

After whatever amount the baby has been able to take with the Playtex™ system, they should not be forced to continue training against their will. The remaining amount can be offered by finger feeding while the baby is still actively searching; once they refuses the feed, or falls asleep, the remainder is administered via feeding tube.

The time from admission to tube weaning, which may not take place until after discharge, usually corresponds to the child’s age at admission for plate treatment—although, of course, among other factors, the individual anatomy plays a role here as well.

## 5. Discussion and Conclusions

This review has outlined detailed practical aspects of how to use the TPP, intended to help with the further distribution of information on this highly effective, minimally invasive, and well-tolerated treatment to other teams considering its implementation. In contrast to surgical techniques such as MDO, it takes advantage of the growth potential of the mandible seen in infants with RS, so that we can indeed demonstrate catch-up growth that occurs during [17] and even after TPP treatment [18]. Indeed, a recent meta-analysis concluded that the latter appears as effective in terms of airway- and feeding-related outcomes as MDO [19]. While we have used conventional techniques in the dental lab for many years, these have recently been replaced by a semi-digital workflow using CAD/CAM techniques. This has been described elsewhere by us [20,21,22,23] and other groups [24] and is therefore not reported on in more detail here. With this new workflow, it has also become comparatively easy to produce a second plate once the infant has outgrown his/her first plate. It has greatly facilitated TPP treatment and might also make it easier for other centers to adopt our approach. It may even lead the way to telehealth, by enabling remote learning, supervision, or even designing an appropriate appliance remotely. It should be kept in mind, however, that this treatment requires an interdisciplinary team consisting of neonatologists competent in upper airway endoscopy, orthodontists, neonatal nurses, speech therapists, and craniofacial surgeons. It is also fraught with the risk of traumatizing the infants’ airway, which is why we have started to offer training courses for teams seriously interested in adopting our approach. Its successful adoption by various centers around the world [25,26,27,28,29,30] makes us confident that this is possible.

## Data Availability

No new data were created or analyzed in this study. Data sharing is not applicable to this article.
